# Usefulness of the cachexia index as a prognostic indicator for patients with gastric cancer

**DOI:** 10.1002/ags3.12669

**Published:** 2023-03-15

**Authors:** Keigo Nakashima, Koichiro Haruki, Teppei Kamada, Junji Takahashi, Yuichi Nakaseko, Hironori Ohdaira, Kenei Furukawa, Yutaka Suzuki, Toru Ikegami

**Affiliations:** ^1^ Department of Surgery International University of Health and Welfare Hospital Tochigi Japan; ^2^ Division of Hepatobiliary and Pancreatic Surgery, Department of Surgery The Jikei University School of Medicine Tokyo Japan

**Keywords:** cachexia, gastric cancer, neutrophil‐to‐lymphocyte ratio, serum albumin

## Abstract

**Aim:**

Cachexia is associated with the morbidity and mortality of cancer patients. The cachexia index (CXI) is a novel biomarker of cachexia associated with the prognosis for certain cancers. This study analyzed the relationship between CXI with long‐term outcomes of gastric cancer patients.

**Methods:**

We included 175 gastric cancer patients who underwent curative gastrectomy at our hospital between January 2011 and October 2019. The CXI was calculated using skeletal muscle index, serum albumin level, and neutrophil‐to‐lymphocyte ratio. The prognostic value of CXI was investigated by univariate and multivariate Cox hazard regression models adjusting for potential confounders.

**Results:**

In the multivariate analyses, tumor location (hazard ratio [HR], 0.23; 95% confidence interval [CI], 0.11–0.49; *p* < 0.01), disease stage (HR, 15.4; 95% CI, 4.18–56.1; *p* < 0.01), and low CXI (HR, 2.97; 95% CI, 1.01–8.15; *p* = 0.03) were independent and significant predictors of disease‐free survival. Disease stage (HR, 9.88; 95% CI, 3.53–29.1; *p* < 0.01) and low CXI (HR, 4.07; 95% CI, 1.35–12.3; *p* < 0.01) were independent and significant predictors of overall survival. The low CXI group had a lower body mass index (*p* = 0.02), advanced disease stage (*p* = 0.034), and a lower prognostic nutritional index (*p* < 0.01).

**Conclusions:**

Cachexia index is associated with a poor prognosis for gastric cancer, suggesting the utility of comprehensive assessment using nutritional, physical, and inflammatory status.

## Graphical abstract

Cachexia index, a recently defined parameter can be a prognostic predictor for gastric cancer patients. Our results suggest that inflammatory status and muscle mass are associated with tumor recurrence and progression in gastric cancer.

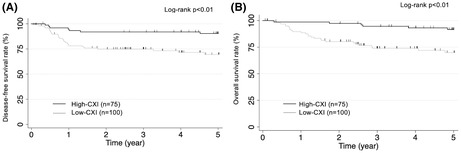

## 1 INTRODUCTION

Gastric cancer is the fourth most prevalent malignancy worldwide and the second leading cause of cancer‐related deaths.^1^ Although advances in treatment have improved the prognosis of early‐stage cancer,^2^ advanced cancer still has a high recurrence rate and a poor prognosis.^1^ Therefore, the investigation of preoperative biomarkers to predict therapeutic outcomes is critically important for clinical decision‐making.

Cancer cachexia, a multifactorial syndrome defined as ongoing loss of skeletal muscle mass with or without a decrease in fat mass, is associated with approximately 30% of cancer‐related deaths.^3^ It is also characterized by systemic inflammation, progressive weight loss, and malnutrition that cannot be fully evaluated by one of these components due to its complexity. Recently, a new parameter called the cachexia index (CXI)^4^, ^5^ has been proposed and is gaining recognition as a potentially comprehensive measure of the state of cachexia. The CXI is composed of parameters such as the skeletal muscle index (SMI), serum albumin level, and neutrophil‐to‐lymphocyte ratio (NLR). Further, the CXI has been reported to be able to predict the prognosis for several malignancies.^5^, ^6^ However, the utility of the CXI as a prognostic factor in gastric cancer patients has not been reported. Therefore, we here investigated the prognostic value of the CXI in gastric cancer patients.

## 2 PATIENTS AND METHODS

### 2.1 Patients

In this study, all patients who underwent laparoscopic or robotic gastrectomy at the Department of Surgery, International University of Health and Welfare Hospital between January 2011 and October 2019 were included in the study. Patients with remnant gastric cancer and locally advanced unresectable tumors were excluded and the remaining 175 eligible patients were enrolled in this study. We retrospectively collected clinical and laboratory data, including computed tomography (CT) scan results, using the hospital's electronic patient record system.

This study was approved by the appropriate Research Ethics Committee at the International University of Health and Welfare Hospital (approval no. 22‐B‐7) and was conducted in accordance with the Declaration of Helsinki. The requirement for informed consent was waived considering the study design and anonymization of data.

### 2.2 Treatment and follow‐up

Distal or total gastrectomy was performed as the standard procedure. Gastrectomy was performed with D1 plus lymph node dissection for early gastric cancer, and D2 lymph node dissection for advanced cancer. Tumor‐node‐metastasis (TNM) classification was quoted from the latest version of the Japanese Classification of Gastric Carcinoma (the 5th edition).^7^ Postoperative complications were defined as grade III of Clavien–Dindo classification or higher that occurred within 30 postoperative days. Patients with stage II or III received adjuvant chemotherapy according to the Japanese gastric cancer treatment guidelines,^8^ if the general condition was judged to be tolerated. The patients were followed up every 3 months to check for the recurrence by performing blood tests, including those for tumor markers, after the operation. Moreover, CT was performed at least every 6 months after the operation.

### 2.3 CXI and nutritional assessment

The CXI was calculated as follows: SMI × serum albumin level (g/dL) / NLR.^5^, ^6^ In this equation, based on a previously described method, to calculate the SMI we used the minor and major diameters of the right iliopsoas muscle of the L3 vertebra level, which were measured using preoperative CT.^6^ The SMI was calculated as follows: iliopsoas minor axis (cm) × major axis (cm) × / height squared (cm^2^/ m^2^).^9^, ^10^ The cutoff values of SMI and CXI were determined by a receiver‐operating characteristic curve using the survival status at the 3‐year follow‐up in strata of sex, considering the differences in the skeletal muscle between males and females. According to the cutoff values, all the patients were divided into the low and high CXI groups. The NLR was defined as the neutrophil count divided by the lymphocyte count.^11^, ^12^ The Prognostic Nutritional Index (PNI) was calculated as 10 × serum albumin level (g/dL) + 0.005 × lymphocyte count.^13^ The cutoff values of albumin and PNI were determined by a receiver‐operating characteristic curve using the survival status at the 3‐year follow‐up.

### 2.4 Statistical analysis

All statistical analyses were performed using STATA version 14 (Stata Corporation). All *p*‐values were two‐sided, and we used the two‐sided α level of 0.05. The data are expressed as medians, ranges, and ratios. Continuous and categorical variables were compared using the Mann–Whitney *U*‐test or chi‐square test, as appropriate. The endpoint, which was overall survival (OS), was defined as time from the date of surgery until the date of death from any cause or the last follow‐up date for the living patients. Disease‐free survival (DFS) was defined as time from the date of surgery to the date of gastric cancer relapse, the last follow‐up date, or death.

Univariable and multivariable Cox proportional hazards regression models were conducted to estimate hazard ratio (HR) and 95% confidence intervals (CIs) for DFS and OS. A multivariate analysis was performed for factors with *p* < 0.05 in the univariate analysis. The Kaplan–Meier method was used to estimate cumulative survival probabilities, and the differences between groups were compared using the log‐rank test.

## 3 RESULTS

### 3.1 Patients' characteristics

The CXI cutoff values were set at 8.01 for men and 5.85 for women by ROC analyses. Using the cutoff values, 100 patients (57%) were allocated to the low CXI group, and 75 patients (43%) were allocated to the high CXI group. A comparison of the patient characteristics of these two groups is summarized in Table 1. The low CXI group was older (*p* < 0.01) and had a lower body mass index (BMI) (*p* = 0.02), advanced N factor (*p* = 0.026), and advanced disease stage (*p* = 0.034). In terms of body composition, the low CXI group had a lower PNI (*p* < 0.01), NLR (*p* < 0.01), serum albumin level (*p* = 0.017), and SMI (*p* < 0.01). The patient backgrounds of both groups in the early stage (stage I) and advanced stage (stage II and III) are summarized in Tables S1‐S2. The low CXI group had a lower PNI (*p* < 0.01), SMI (*p* < 0.01), serum albumin level (*p* < 0.01), and higher NLR (*p* < 0.01) in the early stage, and lower PNI (*p* < 0.01), SMI (*p* = 0.02), serum albumin level (*p* = 0.02), and higher NLR (*p* < 0.01) in the advanced stage.

**TABLE 1:** Baseline characteristics according to the cachexia index (CXI) in 175 gastric cancer patients

Variables	Total	High‐CXI^a^	Low‐CXI	*p*‐value
		n (%) or median (range)		
Patients	175	75 (43%)	100 (57%)	
Age, years	70 (38–92)	66 (38–84)	72 (39–92)	<0.01
Sex				
Male	119 (68%)	52 (69%)	67 (67%)	0.74
Female	56 (32%)	23 (31%)	33 (33%)	
Body mass index, kg/m^2^	20.3 (13.3–29.3)	21.0 (13.3–29.3)	19.8 (13.6–29.1)	0.02
Tumor location				
Upper part	42 (24%)	22 (29%)	20 (20%)	0.25
Middle part	47 (27%)	21 (28%)	26 (26%)	
Lower part	86 (49%)	32 (43%)	54 (54%)	
CEA, ng/mL	2.4 (0.5–51.6)	2.6 (0.6–47.8)	2.4 (0.5–51.6)	0.86
CA19‐9, U/mL	8.3 (0.8–455)	8.0 (0.8–357)	8.5 (1–455)	0.57
Surgical procedure				
Distal gastrectomy	109 (62%)	44 (59%)	65 (65%)	0.39
Total gastrectomy	66 (38%)	31 (41%)	35 (35%)	
Lymph node dissection				
D1	14 (8.0%)	5 (6.7%)	9 (9.0%)	0.74
D1+	82 (47%)	34 (45%)	48 (48%)	
D2	79 (45%)	36 (48%)	43 (43%)	
Operative time, min	331 (173–625)	334 (178–576)	330 (173–625)	0.72
Intraoperative blood loss, mL	25 (5–1650)	30 (5–1650)	17.5 (5–500)	0.17
Postoperative complication, yes	29 (17%)	11 (15%)	18 (18%)	0.56
Adjuvant chemotherapy, yes	60 (35%)	18 (25%)	42 (42%)	0.02
T factor				
T1	97 (55%)	50 (67%)	47 (47%)	0.06
T2	14 (8.0%)	6 (8.0%)	8 (8.0%)	
T3	38 (22%)	11 (15%)	27 (27%)	
T4	26 (15%)	8 (11%)	18 (18%)	
N factor				
N0	113 (65%)	57 (76%)	56 (56%)	0.026
N1	27 (15%)	10 (13%)	17 (17%)	
N2	13 (7.4%)	4 (5.3%)	9 (9.0%)	
N3	22 (13%)	4 (18%)	18 (82%)	
TNM stage				
I	99 (57%)	50 (67%)	49 (49%)	0.034
II	38 (22%)	15 (20%)	23 (23%)	
III	38 (2%)	10 (13%)	28 (28%)	
Histopathology				
Well differentiated adenocarcinoma	47 (27%)	22 (29%)	25 (25%)	0.39
Moderately differentiated adenocarcinoma	57 (33%)	22 (29%)	35 (35%)	
Poorly differentiated adenocarcinoma	45 (26%)	19 (25%)	26 (26%)	
Signet‐ring cell adenocarcinoma	19 (11%)	8 (11%)	11 (11%)	
Mucinous adenocarcinoma	4 (1.7%)	1 (1.3%)	3 (3.0%)	
Papillary adenocarcinoma	3 (1.7%)	3 (4.0%)	0 (0.0%)	
Microvascular invasion, yes	64 (37%)	19 (25%)	45 (45%)	<0.01
Microlymphatic invasion, yes	77 (44%)	24 (32%)	53 (53%)	<0.01
PNI	49.7 (23.1–66.3)	52.3 (34.7–66.3)	46.6 (23.1–62.8)	<0.01
NLR	1.94 (0.43–8.52)	1.37 (0.43–3.44)	2.37 (1.14–8.52)	<0.01
Albumin, g/dL	4.0 (1.9–4.8)	4.2 (2.7–4.8)	3.9 (1.9–4.8)	<0.01
SMI, cm^2^/m^2^	3.09 (0.96–10.7)	3.67 (1.55–10.7)	2.75 (0.96–5.29)	<0.01

*Abbreviations*: CEA, carcinoembryonic antigen; CA19‐9, carbohydrate antigen 19–9; PNI, Prognostic nutritional index; NLR, Neutrophil‐to‐lymphocyte ratio; SMI, Skeletal muscle mass index.

^a^ The CXI cutoff values were set at 8.01 for men and 5.85 for women.

### 3.2 Prognostic significance of CXI in gastric cancer patients

**FIGURE 1:**
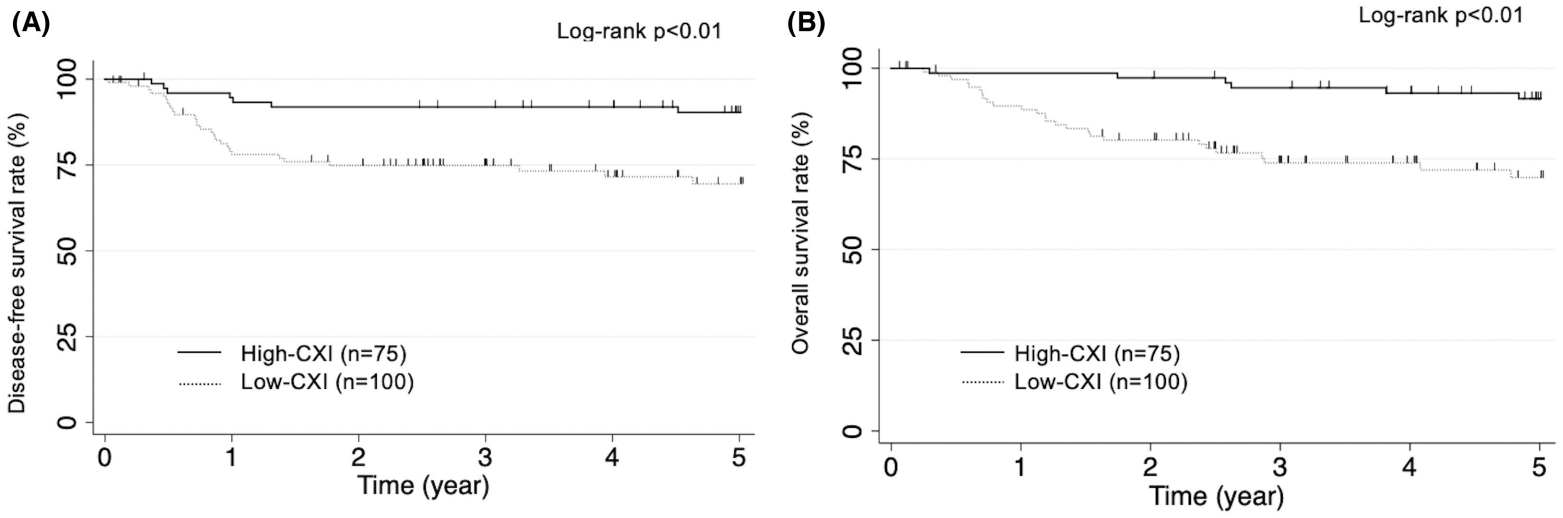
Kaplan–Meier curve of disease‐free (A) and overall (B) survival according to the status of cachexia index after gastrectomy for gastric cancer. Low cachexia index was significantly associated with worse disease‐free (*p* < 0.01) and overall survival (*p* < 0.01)

Kaplan–Meier curves showed that low CXIs were significantly associated with worse DFS (*p* < 0.01) and OS (*p* < 0.01) (Figure 1).

The cutoff values of SMI were set at 3.53 for men and 3.04 for women, respectively. The cutoff values of albumin, PNI, and NLR were set at 4.0, 45.6, and 1.71, respectively, determined by ROC curve. The univariate analysis of DFS for all patients indicated that tumor location (*p* = 0.026), advanced stage (*p* < 0.01), microvascular invasion (*p* < 0.01), microlymphatic invasion (*p* < 0.01), low SMI (*p* = 0.04), high NLR (*p* = 0.01), and low CXI (*p* < 0.01) were significant prognostic factors (Table 2). The multivariate analysis showed that tumor location (HR, 0.23; 95% CI, 0.11–0.49; *p* = *p* < 0.01), advanced disease stage (HR, 15.4; 95% CI, 4.18–56.1; *p* < 0.01), and low CXI (HR, 2.97; 95% CI, 1.01–8.15; *p* = 0.03) were independently associated with unfavorable outcomes (Table 2).

**TABLE 2:** Univariate and multivariate analysis for disease‐free survival in patients with gastric cancer

Variables	Univariate analysis		Multivariate analysis	
	HR (95% CI)	*p*‐value	HR (95% CI)	*p*‐value
Age, ≥70 years	1.39 (0.73–2.67)	0.32		
Sex, male	1.73 (0.79–3.80)	0.17		
Tumor location, upper part	0.46 (0.24–0.91)	0.026	0.23 (0.11–0.49)	<0.01
CEA, ≥5.0 ng/mL	0.32 (0.78–1.35)	0.12		
CA19‐9, ≥37.0 U/mL	1.23 (0.43–3.46)	0.70		
Albumin, <4.0 g/dL	1.09 (0.57–2.09)	0.80		
Postoperative complication, yes	1.71 (0.78–3.74)	0.18		
Stage, II or III	21.8 (6.67–71.2)	<0.01	15.4 (4.18–56.1)	<0.01
Histological type, undifferentiated	1.88 (0.98–3.59)	0.055		
Microvascular invasion, yes	5.49 (2.69–11.17)	<0.01	1.38 (0.65–2.94)	0.40
Microlymphatic invasion, yes	6.41 (2.92–14.09)	<0.01	1.71 (0.72–4.08)	0.22
SMI, low^a^	2.12 (1.04–4.31)	0.04	1.41 (0.66–3.03)	0.37
PNI, <45.6	1.81 (0.92–3.56)	0.09		
NLR, ≥1.71	2.65 (1.26–5.55)	0.01	0.93 (0.37–2.32)	0.88
CXI, low^b^	3.66 (1.65–8.11)	<0.01	2.97 (1.01–8.15)	0.03

*Abbreviations*: HR, hazard ratio; CI, confidence interval; CEA, carcinoembryonic antigen; CA19‐9, carbohydrate antigen 19–9; SMI, skeletal muscle mass index; PNI, prognostic nutritional index; NLR, neutrophil‐to‐lymphocyte ratio; CXI, cachexia index.

^a^ The SMI cutoff values were set at 3.53 for men and 3.04 for women.

^b^ The CXI cutoff values were set at 8.01 for men and 5.85 for women.

**FIGURE 2:**
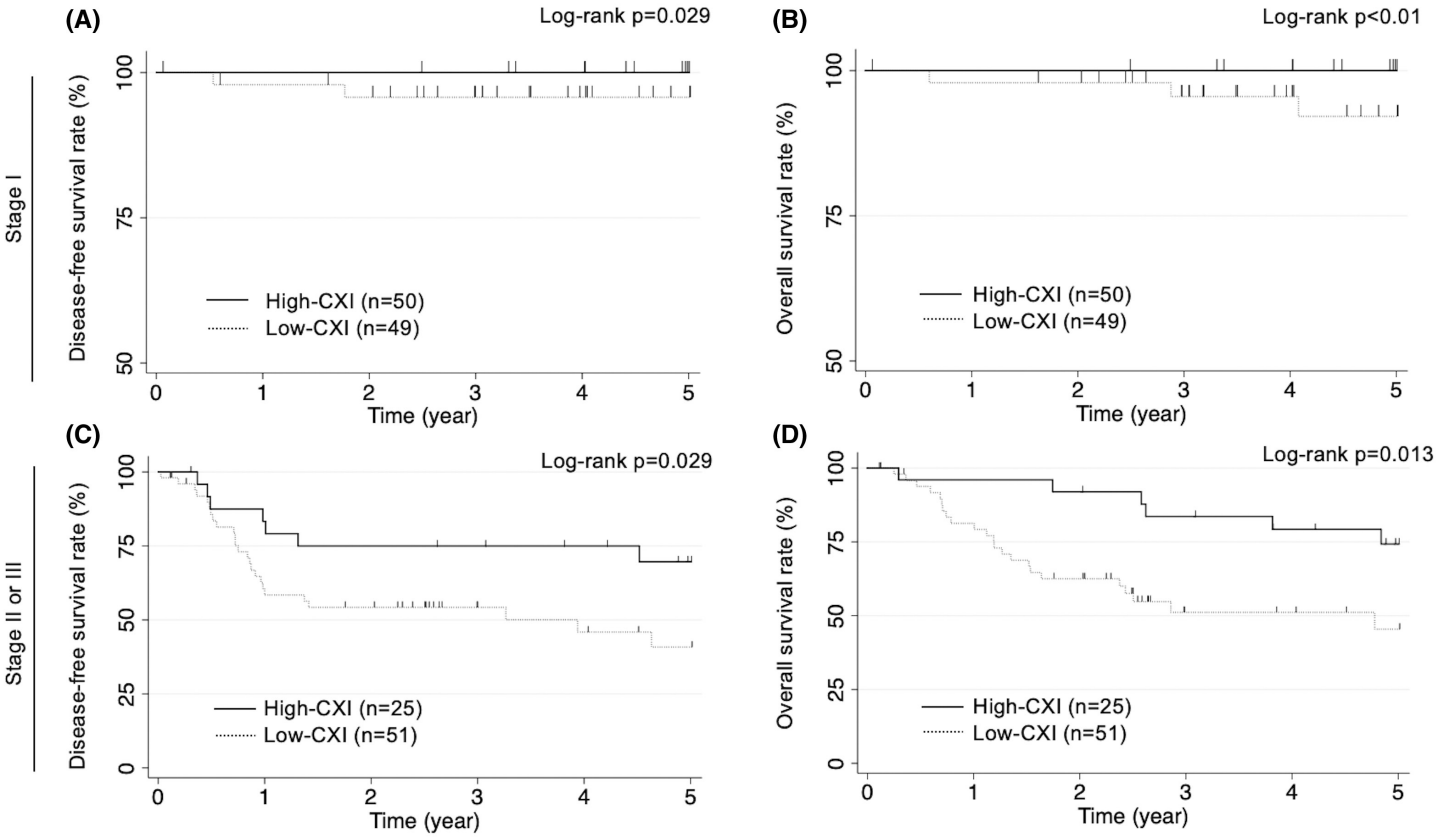
Kaplan–Meier curve of disease‐free and overall survival according to the status of cachexia index in the subgroup of stage I (A and B) and stage II or III (C and D) after gastrectomy for gastric cancer. Low cachexia index was significantly associated with worse disease‐free and overall survival regardless of the disease stage (stage I, and stage II or III) (all *p* < 0.05)

The univariate analysis of OS for all patients indicated that older age (*p* = 0.018), postoperative complication (*p* = 0.044), advanced disease stage (*p* < 0.01), microvascular invasion (*p* < 0.01), microlymphatic invasion (*p* < 0.01), low SMI (*p* < 0.01), high NLR (*p* = 0.02), and low CXI (*p* < 0.01) were significant prognostic factors (Table 3). The multivariate analysis revealed that advanced disease stage (HR, 9.88; 95% CI, 3.35–29.1; *p* < 0.01) and low CXI (HR, 4.07; 95% CI, 1.35–12.3; *p* = 0.01) were independent and significant predictors of OS (Table 3). We assessed the association of the CXI status with DFS and OS in subgroups of disease stage. The low CXI was significantly associated with worse DFS and OS regardless of the disease stage (stage I and stage II or III) (all *p* < 0.05, Figure 2).

**TABLE 3:** Univariate and multivariate analysis for overall survival in patients with gastric cancer

Variables	Univariate analysis		Multivariate analysis	
	HR (95% CI)	*p*‐value	HR (95% CI)	*p*‐value
Age, ≥70 years	2.23 (1.14–4.33)	0.018	1.84 (0.85–3.99)	0.12
Sex, male	1.60 (0.75–3.38)	0.22		
Tumor location, upper part	0.59 (0.30–1.18)	0.14		
CEA, ≥5.0 ng/mL	1.08 (0.45–2.58)	0.87		
CA19‐9, ≥37.0 U/mL	0.97 (0.30–3.16)	0.96		
Albumin, <4.0 g/dL	1.03 (0.54–1.98)	0.93		
Postoperative complication, yes	2.18 (1.02–4.69)	0.044	1.50 (0.67–3.38)	0.32
Stage, II or III	11.8 (4.86–28.7)	<0.01	9.88 (3.35–29.1)	<0.01
Histological type, undifferentiated	1.69 (0.89–3.20)	0.11		
Microvascular invasion, yes	3.76 (1.94–7.29)	<0.01	0.82 (0.37–1.82)	0.63
Microlymphatic invasion, yes	4.79 (2.36–9.73)	<0.01	1.43 (0.60–3.40)	0.42
SMI, low^a^	3.11 (1.47–6.60)	<0.01	1.28 (0.54–3.07)	0.58
PNI, <45.6	1.52 (0.75–3.11)	0.24		
NLR, ≥1.71	2.32 (1.15–4.65)	0.02	0.76 (0.29–1.95)	0.57
CXI, low^b^	4.63 (2.08–10.3)	<0.01	4.07 (1.35–12.3)	0.01

*Abbreviations*: HR, hazard ratio; CI, confidence interval; CEA, carcinoembryonic antigen; CA19‐9, carbohydrate antigen 19–9; SMI, skeletal muscle mass index; PNI, prognostic nutritional index; NLR, neutrophil‐to‐lymphocyte ratio; CXI, cachexia index.

^a^ The SMI cutoff values were set at 3.53 for men and 3.04 for women.

^b^ The CXI cutoff values were set at 8.01 for men and 5.85 for women.

## 4 DISCUSSION

In this study, we found that a low CXI was associated with worse survival of gastric cancer patients. Notably, this survival association was consistent in both early and advanced stage of gastric cancer. Our results suggest the importance of assessing cancer cachexia even in the early stage. Moreover, CXI may help to evaluate cancer cachexia comprehensively and provide better predictive value for long‐term outcomes.

Cancer cachexia is a syndrome characterized by reduction of muscle mass and anorexia, which is frequently seen in patients with advanced cancer. It has been proposed that inflammatory cytokines produced by tumors have a profound effect on cachexia. An increase in interleukin‐6 or tumor necrosis factor‐α causes loss of appetite, muscle atrophy, and increased energy consumption that leads to cachexia. Several studies have suggested that myokines produced by skeletal muscles might have anti‐cancer effects.^14^, ^15^ Therefore, decreased myokine levels caused by cancer cachexia might be associated with a poor prognosis. Cachexia has also been associated with reduced chemotherapeutic effects, increased side effects, and treatment interruptions, resulting in poor outcome in cancer patients.^16^, ^17^ In this study, 49% of early gastric cancer patients showed low CXI. Evidence suggests that sarcopenia and high systemic inflammatory response (i.e. high NLR) have been observed in early gastric cancer and associated with worse survival.^11^, ^18^ In the previous reports, 12% of the early gastric cancer patients had sarcopenia,^18^ while 55% of those patients showed high NLR.^12^ Since chronic inflammation has been associated with the carcinogenesis of gastric cancer,^19^ early gastric cancer patients might have sarcopenia and high inflammatory status caused by chronic inflammation. Therefore, low CXI, represented by sarcopenia and high NLR, can potentially occur in early gastric cancer patients. The assessment of CXI in early‐stage gastric cancer can identify patients who need nutritional and physical interventions.

Although the understanding of cachexia has progressed over the past decades, a helpful index and its clinical practice are still lacking. The CXI is a new index that includes sarcopenia, inflammation, and malnutrition, each of which has been reported to be a prognostic factor of gastric cancer.^11^, ^12^, ^13^, ^20^, ^21^, ^22^ CXI may help to detect the state of cachexia as a condition that should not be overlooked. Given our findings where CXI had the highest HRs for disease‐free and overall survivals among each component of CXI and nutritional parameter, we considered that it is helpful to know whether CXI would be a better prognostic indicator for gastric cancer compared with the known factors including PNI and NLR. Further studies are needed to elucidate what parameter is the best to predict the prognosis in gastric cancer.

Inflammation caused by cancer cachexia is important in initiating malignancy through the exposure of proinflammatory cytokines and sustained activation of signaling pathways. Inflammatory cytokines can result in immune system dysfunction and accelerate tumor development, progression, and metastasis. Evidence suggests that inflammation promotes angiogenesis and induces infiltration of myeloid‐derived suppressor cells and tumor‐associated macrophages, resulting in the creation of a microenvironment favorable for tumor growth, which plays a pivotal role in promoting tumor cell invasion and metastasis.^23^, ^24^, ^25^ Taken together, inflammation due to inflammatory cytokines may promote cachexia itself as well as cancer development, leading to poor outcomes in gastric cancer patients.^19^

In the aging population, more patients with low CXI are expected to require surgical intervention. Comprehensive knowledge of cancer and cachexia is essential for the development of medical strategies for gastric cancer patients. Our findings emphasize that it is important to consider both the clinical and patient factors to maximize the quality of life and improve survival in cancer patients. Patients with low CXI may need intensive nutritional and exercise guidance to improve skeletal muscle mass and prevent malnutrition before surgery. Although nutritional interventions has been less effective in the presence of malnutrition, loss of muscle mass, or sarcopenia with high inflammatory cytokines,^26^ combination of nutritional and physical intervention may have possibility to improve these status compared with nutritional or physical intervention alone.^27^ A study indicated that among sarcopenia patients with gastric cancer, 18% of those patients was improved to be nonsarcopenic after 3 weeks of preoperative exercise and nutritional support.^28^ Moreover, another study showed that preoperative nutrition and exercise intervention in patients with gastric cancer have improved NLR, resulting in decreased secretion of cytokines and chemokines, which play important roles in cancer progression.^29^ Since NLR and sarcopenia are the components of the CXI, early nutritional intervention and rehabilitation can potentially improve the CXI, leading to a better prognosis. Therefore, our findings suggest that evaluation of CXI can be useful to identify patients who need nutritional and physical intervention even in patients with early‐stage gastric cancer. Further studies are warranted to investigate whether early prevention or improvement of cancer cachexia contributes to improved survival in gastric cancer patients.

We acknowledge the potential limitation of this study. This study was principally limited by its small sample size and single‐center retrospective design, which might have caused a selection bias. In addition, the cut‐off value of CXI was determined using data from a single center, which may lack generalizability. Therefore, although this is the first study to investigate the relationship between the CXI findings and clinical outcomes in gastric cancer patients, our findings need to be validated by large‐scale studies.

In conclusion, the CXI might be a valuable factor for predicting the DFS and OS of gastric cancer patients. Our results suggest the utility of comprehensive assessment using nutritional, physical, and inflammatory status in gastric cancer patients.

## AUTHOR CONTRIBUTIONS

KN and KH developed the main concept and designed the study. KN, KH, TK, JT, YN, HO, KF, YS, and TI were responsible for acquisition of clinicopathological data. KN and KH performed data analysis, interpretation, and drafted the manuscript. TK, JT, YN, HO, KF, YS, and TI contributed to editing and critical revision for important intellectual content.

## FUNDING INFORMATION

This work was supported by JSPS KAKENHI Grant Number JP21K08805 and by research grants from the Uehara Memorial Foundation and Japanese Foundation for Multidisciplinary Treatment of Cancer.

## CONFLICT OF INTEREST

The authors declare that they have no conflicts of interest.

## ETHICS STATEMENTS

Approval of the research protocol: This study protocol was approved by the Research Ethics Committee at the International University of Health and Welfare Hospital (#22‐B‐7). Patients were given an opportunity to optout of this study through public announcements.

Informed consent: N/A.

Registry and the Registration No. of the study/trial: N/A.

Animal Studies: N/A.

## Supporting information


TABLES S1‐S2.
