# Supplementary material for: Usefulness of the cachexia index as a prognostic indicator for patients with gastric cancer
Source: Ann Gastroenterol Surg. 2023 Mar 15;7(5):733–40. doi: 10.1002/ags3.12669 (PMC10472360; doi:10.1002/ags3.12669)
Supplement: Supplementary file 1 — TABLES S1‐S2. [file AGS3-7-733-s001.docx]

**Supplementary Table 1.** Baseline characteristics according to the cachexia index (CXI) in early gastric cancer patients (Stage I)

| Variables | | Total | High-CXI | Low-CXI | p-value |
| --- | --- | --- | --- | --- | --- |
|  | |  | n (%) or median (range) | |  |
| Patients | | 99 | 50 (51%) | 49 (49%) |  |
| Age, years | | 69 (39-92) | 66 (40-82) | 70 (39-92) | 0.06 |
| Sex | Male | 67 (68%) | 34 (69%) | 33 (67%) | 0.95 |
|  | Female | 32 (32%) | 16 (31%) | 16 (33%) |  |
| Body mass index, kg/m^2^ | | 21.3 (14.8-29.1) | 21.3 (14.8-28.0) | 20.0 (14.8-29.1) | 0.33 |
| Tumor location | |  |  |  | 0.25 |
| Upper part | | 23 (23%) | 14 (28%) | 9 (18%) |  |
| Middle part | | 26 (26%) | 15 (30%) | 11 (22%) |  |
| Lower part | | 50 (51%) | 21 (42%) | 29 (59%) |  |
| CEA, ng/ml | | 2.5 (0.5-51.6) | 2.6 (0.6-47.8) | 2.5 (0.5-51.6) | 0.94 |
| CA19-9, U/ml | | 8.0 (1.0-357) | 8.0 (1.0-357) | 7.6 (1.0-47.7) | 0.77 |
| Surgical procedure | |  |  |  | 0.03 |
| Distal gastrectomy | | 69 (70%) | 30 (60%) | 39 (80%) |  |
| Total gastrectomy | | 30 (30%) | 20 (40%) | 10 (20%) |  |
| Lymph node dissection | |  |  |  | 0.70 |
| D1 | | 6 (6.0%) | 3 (6.0%) | 3 (6.1%) |  |
| D1+ | | 56 (57%) | 30 (60%) | 26 (53%) |  |
| D2 | | 37 (37%) | 17 (34%) | 20 (41%) |  |
| Operative time, min | | 320 (215-576) | 328 (244-576) | 312 (215-483) | 0.13 |
| Intraoperative blood loss, ml | | 20 (5-373) | 30 (5-373) | 10 (5-150) | <0.01 |
| Postoperative Complication, yes | | 14 (14%) | 6 (12%) | 8 (16%) | 0.54 |
| Adjuvant chemotherapy, yes | | 60 (35%) | 18 (25%) | 42 (42%) | 0.02 |
| T factor | |  |  |  | 0.53 |
| T1 | | 93 (4%) | 48 (96%) | 45 (92%) |  |
| T2 | | 6 (6.0%) | 2 (4.0%) | 4 (8.0%) |  |
| T3 | | 0 (0.0%) | 0 (0.0%) | 0 (0.0%) |  |
| T4 | | 0 (0.0%) | 0 (0.0%) | 0 (0.0%) |  |
| N factor | |  |  |  | 0.43 |
| N0 | | 90 (91%) | 47 (94%) | 43 (88%) |  |
| N1 | | 9 (9.0%) | 3 (6.0%) | 6 (12%) |  |
| N2 | | 0 (0%) | 0 (0.0%) | 0 (0.0%) |  |
| N3 | | 0 (0%) | 0 (0.0%) | 0 (0.0%) |  |
| TNM stage | |  |  |  |  |
| I | | 99 | 50 (51%) | 49 (49%) |  |
| II | | 0 (0%) | 0 (0.0%) | 0 (0.0%) |  |
| III | | 0 (0%) | 0 (0.0%) | 0 (0.0%) |  |
| Histopathology | |  |  |  | 0.72 |
| Well differentiated adenocarcinoma | | 38 (38%) | 19 (38%) | 19 (39%) |  |
| Moderately differentiated adenocarcinoma | | 31 (31%) | 14 (28%) | 17 (35%) |  |
| Poorly differentiated adenocarcinoma | | 17 (17%) | 10 (20%) | 7 (14%) |  |
| Signet-ring cell adenocarcinoma | | 11 (11%) | 5 (10%) | 6 (12%) |  |
| Mucinous adenocarcinoma | | 1 (1.0%) | 1 (2.0%) | 0 (0.0%) |  |
| Papillary adenocarcinoma | | 1 (1.0%) | 1 (2.0%) | 0 (0.0%) |  |
| Microvascular invasion, yes | | 14 (14%) | 6 (12%) | 8 (16%) | 0.54 |
| Microlymphatic invasion, yes | | 20 (20%) | 9 (18%) | 11 (22%) | 0.58 |
| PNI | | 50.4 (23.1-66.3) | 53.0 (34.7-61.5) | 48.5 (32.3-55.9) | <0.01 |
| NLR | | 1.77 (0.43-5.89) | 1.26 (0.43-2.81) | 2.36 (1.29-5.89) | <0.01 |
| Albumin, g/dl | | 4.1 (2.4-4.8) | 4.2 (2.7-4.8) | 3.9 (2.4-4.8) | <0.01 |
| SMI, cm^2^/m^2^ | | 3.39 (1.46-7.3) | 3.82 (1.81-7.3) | 2.70 (1.46-5.29) | <0.01 |

*The CXI cutoff values were set at 8.01 for men and 5.85 for women.

Abbreviations: CEA, carcinoembryonic antigen; CA19-9, carbohydrate antigen 19-9; PNI, Prognostic nutritional index; NLR, Neutrophil-to-lymphocyte ratio; SMI, Skeletal muscle mass index.

**Supplementary Table 2.** Baseline characteristics according to the cachexia index (CXI) in advanced gastric cancer patients (Stage II, III)

| Variables | | Total | High-CXI | Low-CXI | p-value |
| --- | --- | --- | --- | --- | --- |
|  | |  | n (%) or median (range) | |  |
| Patients | | 76 | 25 (33%) | 51 (67%) |  |
| Age, years | | 72 (38-92) | 67 (38-84) | 75 (57-92) | <0.01 |
| Sex | Male | 24 (32%) | 7 (28%) | 17 (33%) | 0.64 |
|  | Female | 52 (68%) | 18 (72%) | 34 (67%) |  |
| Body mass index, kg/m^2^ | | 19.4 (13.3-29.3) | 20.1 (13.3-29.3) | 19.3 (13.6-28.1) | 0.21 |
| Tumor location | |  |  |  | 0.61 |
| Upper part | | 19 (25%) | 8 (32%) | 11 (22%) |  |
| Middle part | | 21 (28%) | 6 (24%) | 15 (29%) |  |
| Lower part | | 36 (47%) | 11 (44%) | 25 (49%) |  |
| CEA, ng/ml | | 2.4 (0.6-32.1) | 2.7 (0.6-5.5) | 2.2 (0.8-32.1) | 0.69 |
| CA19-9, U/ml | | 8.5 (0.8-455) | 6.3 (0.8-41.2) | 9.1 (1-455) | 0.22 |
| Surgical procedure | |  |  |  | 0.68 |
| Distal gastrectomy | | 40 (53%) | 14 (56%) | 26 (51%) |  |
| Total gastrectomy | | 36 (47%) | 11 (44%) | 25 (49%) |  |
| Lymph node dissection | |  |  |  | 0.03 |
| D1 | | 8 (11%) | 2 (8.0%) | 6 (12%) |  |
| D1+ | | 26 (34%) | 4 (16%) | 22 (43%) |  |
| D2 | | 42 (55%) | 19 (79%) | 23 (45%) |  |
| Operative time, min | | 351 (173-625) | 340 (178-568) | 360 (173-625) | 0.72 |
| Intraoperative blood loss, ml | | 45 (5-1650) | 40 (5-1650) | 50 (5-500) | 0.87 |
| Postoperative Complication, yes | | 15 (20%) | 5 (20%) | 10 (20%) | 0.97 |
| Adjuvant chemotherapy, yes | | 57 (75%) | 18 (72%) | 39 (76%) | 0.67 |
| T factor | |  |  |  | 0.73 |
| T1 | | 4 (5.3%) | 2 (8.0%) | 2 (4.0%) |  |
| T2 | | 9 (12%) | 4 (16%) | 5 (9.8%) |  |
| T3 | | 37 (49%) | 11 (44%) | 26 (51%) |  |
| T4 | | 26 (34%) | 8 (32%) | 18 (35%) |  |
| N factor | |  |  |  | 0.37 |
| N0 | | 23 (30%) | 10 (40%) | 13 (25%) |  |
| N1 | | 19 (25%) | 7 (28%) | 12 (24%) |  |
| N2 | | 13 (17%) | 4 (16%) | 9 (18%) |  |
| N3 | | 21 (28%) | 4 (16%) | 17 (33%) |  |
| TNM stage | |  |  |  | 0.22 |
| I | | 0 (0.0%) | 0 (0.0%) | 0 (0.0%) |  |
| II | | 38 (50%) | 15 (60%) | 23 (45%) |  |
| III | | 38 (50%) | 10 (40%) | 28 (55%) |  |
| Histopathology | |  |  |  | 0.34 |
| Well differentiated adenocarcinoma | | 9 (12%) | 3 (12%) | 6 (12%) |  |
| Moderately differentiated adenocarcinoma | | 26 (34%) | 8 (32%) | 18 (35%) |  |
| Poorly differentiated adenocarcinoma | | 28 (37%) | 9 (36%) | 19 (37%) |  |
| Signet-ring cell adenocarcinoma | | 8 (11%) | 3 (12%) | 5 (9.8%) |  |
| Mucinous adenocarcinoma | | 3 (4.0%) | 0 (0.0%) | 3 (5.9%) |  |
| Papillary adenocarcinoma | | 2 (2.6%) | 2 (8.0%) | 0 (0.0%) |  |
| Microvascular invasion, yes | | 50 (66%) | 13 (52%) | 37 (73%) | <0.01 |
| Microlymphatic invasion, yes | | 57 (75%) | 15 (60%) | 42 (82%) | 0.03 |
| PNI | | 47.5 (23.1-66.3) | 51.2 (38.6-66.3) | 45.1 (23.1-62.8) | <0.01 |
| NLR | | 2.08 (0.43-8.52) | 1.47 (0.43-3.44) | 2.38 (1.14-8.52) | <0.01 |
| Albumin, g/dl | | 3.9 (1.9-4.8) | 4.1 (3.1-4.8) | 3.8 (1.9-4.7) | 0.02 |
| SMI, cm^2^/m^2^ | | 2.94 (0.96-10.7) | 3.40 (1.55-10.7) | 2.76 (0.96-5.29) | 0.02 |

*The CXI cutoff values were set at 8.01 for men and 5.85 for women.

Abbreviations: CEA, carcinoembryonic antigen; CA19-9, carbohydrate antigen 19-9; PNI, Prognostic nutritional index; NLR, Neutrophil-to-lymphocyte ratio; SMI, Skeletal muscle mass index.
